# *Angiopteris weimingii* (Marattiaceae): a new endangered fern species from southern Yunnan, China, revealed by morphology and phylogeny

**DOI:** 10.3897/phytokeys.270.179399

**Published:** 2026-02-02

**Authors:** Li-Ju Jiang, Jing Zhao, Dan-Ni Ma, Jian-Wu Li, Xin-Mao Zhou, Jia-Guan Wang, Zhao-Rong He

**Affiliations:** 1 Centre for Gardening and Horticulture, Xishuangbanna Tropical Botanical Garden, Chinese Academy of Sciences, Menglun, Mengla 666303, Yunnan, China School of Ecology and Environmental Science, Yunnan University Kunming China; 2 School of Ecology and Environmental Science, Yunnan University, Kunming 650504, Yunnan, China School of Life Sciences, Yunnan University Kunming China; 3 Herbarium, Center for Integrative Conservation, Xishuangbanna Tropical Botanical Garden, Chinese Academy of Sciences, Menglun, Mengla 666303, Yunnan, China Xishuangbanna Tropical Botanical Garden, Chinese Academy of Sciences Mengla China; 4 School of Life Sciences, Yunnan University, East Outer Ring Road, Chenggong District, Kunming 650500, Yunnan, China Center for Integrative Conservation, Xishuangbanna Tropical Botanical Garden, Chinese Academy of Sciences Mengla China

**Keywords:** False veins, Marattiaceae, morphology, plastome, spore ornamentation

## Abstract

*Angiopteris weimingii*, a morphologically distinct new species of *Angiopteris* (Marattiaceae) from southern Yunnan, China, is described. Compared with *A. itoi*, the new species can be distinguished by a taller habit (1.2–2.5 m vs. 0.5–1.5 m), a greater number of pinnae pairs (8–13 pairs vs. 4–9 pairs), a caudate tip (1 cm vs. 2–4 cm), the position of sori from the leaf margin (2 mm vs. 3–5 mm), spore color (yellow vs. white), and spore ornamentation (densely verrucate vs. clavate-echinate). Molecular phylogenetic analyses confirm its affinity with the Archangiopteris clade. With a single known population of approximately 30 adults and severe habitat destruction, the species is assessed as Critically Endangered (CR) under IUCN criteria.

## Introduction

Marattiaceae are one of the most ancient lineages of eusporangiate ferns, with extant genera including *Angiopteris* Hoffm., *Christensenia* Maxon, *Danaea* Sm., *Eupodium* J. Sm., *Marattia* Sw., and *Ptisana* Murdock (PPG I 2016). Among these, *Angiopteris* is one of the most species-rich genera and is widely distributed in the Paleotropics, from Madagascar to the South Pacific islands (He and Christenhusz 2013; PPG I 2016; Zhao et al. 2023; POWO 2025). The taxonomy of this genus has undergone significant revisions, particularly regarding the incorporation of the formerly independent genus *Archangiopteris* (Li and Lu 2006; Murdock 2008a, b; He and Christenhusz 2013; PPG I 2016; Zhao et al. 2023). *Archangiopteris* was established by Christ and Giesenhagen (1899) based on diagnostic characters such as simply pinnate fronds, pulvinate stipe articulations, and elongate-linear sori. Despite some uncertainty regarding the robustness of early phylogenetic analyses, molecular phylogenetic studies have consistently confirmed that *Archangiopteris* is nested within *Angiopteris* (Li and Lu 2006; Murdock 2008a, b; He 2009). In addition, putative hybrid species—including *Angiopteris itoi*, *A. involuta*, *A. sparsisora*, and *A. sugongii*—provide critical evidence supporting the broadly defined genus (Ching and Wang 1982; Wu 2002; Wang et al. 2021; Jiang et al. 2025).

Globally, *Angiopteris* comprises approximately 60 species (POWO 2025), with China serving as a center of diversity and hosting more than 30 species (He and Christenhusz 2013). In China, Yunnan Province harbors 19 recorded species (He and Christenhusz 2013; Wang et al. 2020; Wang et al. 2021; Wang et al. 2024; Jiang et al. 2025). This study describes a new species, *Angiopteris weimingii*, discovered in Lvchun County, Honghe Prefecture, Yunnan Province, and named in honor of Professor Wei-Ming Chu for his seminal contributions to the study of lycophytes and ferns. This discovery further enriches the species diversity of *Angiopteris* in Yunnan Province.

## Materials and methods

### Sample collection and morphological analysis

Living material was first collected from Lvchun County in 2008 (collectors: Guo-Da Tao and Jian-Wu Li; XTBG accession number 0359) and is maintained in the nursery of Xishuangbanna Tropical Botanical Garden (XTBG). Based on Tao’s records and description, we conducted field surveys in May 2024 and June 2025, during which one population comprising approximately 30 plants was recorded.

Both cultivated and wild adult individuals were examined. Voucher specimens are deposited in the Pteridophyte Herbarium of Yunnan University (PYU) and the Xishuangbanna Tropical Botanical Garden (XTBG) Herbarium (HITBC) (herbarium acronyms follow Index Herbariorum by Thiers 2024). Gross morphology was examined, and photographs were taken using an SMZ1270 stereo microscope (Nikon, Japan). Spore materials were attached to carbon adhesive tape under an anatomical lens, after which samples were coated with gold using a BAL-TEC SCD 005 Cool Sputter Coater (BAL-TEC AG., Liechtenstein) and visualized using a QUANTA 200 scanning electron microscope (FEI Co., USA) at 25 kV at Yunnan University, Kunming, China.

### Taxonomic sampling, DNA extraction, and sequencing

Three individuals of the new species were sequenced to determine its phylogenetic position. Total genomic DNA was extracted from silica-dried leaf material using the TIANGEN plant genomic DNA extraction kit (TIANGEN Biotech., Beijing, China), following the manufacturer’s protocols, and sequenced at Biomaker Technology Co., Ltd. (Beijing, China) for Illumina sequencing. Paired-end reads of 2 × 150 bp were generated using an Illumina NovaSeq 6000 platform (2G data for each sample). In addition, all 19 available plastomes of *Angiopteris* from GenBank and GenBase were included. Accession numbers for these sequences are provided in Table 1.

**Table 1.** Overview of the plastomes used in this study.

Species	Plastome size (bp)	LSC size (bp)	SSC size (bp)	IR size (bp)	GC content (%)	Voucher	Location	GenBank ID	Reference
***Angiopteris angustifolia*** C. Presl	153,596	89,708	20,536	21,676	35.50	Unknown	Unknown	NC026300	Zhu et al. 2016
***Angiopteris bipinnata*** (Ching) J.M. Camus	153,381	89,709	20,580	21,546	35.40	JLJ-09-0646 (HITBC)	Yunnan, China	PX138845	Jiang et al. 2025
***Angiopteris caudatiformis*** Hieron.	153,137	89,710	20,585	21,421	35.40	JLJ-09-0207 (HITBC)	Yunnan, China	PX138846	Jiang et al. 2025
***Angiopteris chingii*** J.M. Camus	153,134	89,738	20,564	21,416	35.50	JLJ-09-2022 (HITBC)	Yunnan, China	PX138847	Jiang et al. 2025
***Angiopteris chingii*** J.M. Camus	152,551	89,917	20,564	21,035	35.50	YYH16228-1	Yunnan, China	PP056126	Wang et al. 2024
***Angiopteris chingii*** J.M. Camus	152,551	89,929	20,564	21,029	35.50	YYH22077	Yunnan, China	PP056122	Wang et al. 2024
***Angiopteris fokiensis*** Hieron	153,063	89,706	20,585	21,386	35.50	Unknown	China	NC068854	Unknown
***Angiopteris guangdongensis*** Wufeng Chen & Y.H. Yan	153,069	89, 712	20,585	21,386	35.40	YYH24298 (NOCC)	Guangdong, China	C_AA110714	Chen et al. 2025
***Angiopteris guangdongensis*** Wufeng Chen & Y.H. Yan	153,069	89, 712	20,585	21,386	35.40	YYH24298.3(NOCC)	Guangdong, China	C_AA110715	Chen et al. 2025
***Angiopteris guangdongensis*** Wufeng Chen & Y.H. Yan	153,069	89, 712	20,585	21,386	35.40	YYH24298.4 (NOCC)	Guangdong, China	C_AA110716	Chen et al. 2025
***Angiopteris guangdongensis*** Wufeng Chen & Y.H. Yan	153,069	89, 712	20,585	21,386	35.40	YYH24298.5 (NOCC)	Guangdong, China	C_AA110717	Chen et al. 2025
***Angiopteris involuta*** L.J. Jiang & Z.R. He	153,154	89,742	20,565	21,424	35.40	JLJ-09-1591 (PYU)	Yunnan, China	PX138848	Jiang et al. 2025
***Angiopteris itoi*** (W.C. Shieh) J.M. Camus	153,379	89,712	20,585	21,541	35.40	Pan2024-a (PYU)	Taiwan, China	PX138849	Jiang et al. 2025
***Angiopteris itoi*** (W.C. Shieh) J.M. Camus	153,379	89,712	20,585	21,541	35.40	Pan2024-b (PYU)	Taiwan, China	PX138850	Jiang et al. 2025
***Angiopteris latipinna*** (Ching) Z. R. He, W. M. Chu & Christenh	153,597	89,925	20,562	21,555	35.50	YYH16502	Yunnan, China	PP056125	Wang et al. 2024
***Angiopteris nodosipetiolata*** Ting Wang tris, H.F. Chen & Y.H. Yan	152,964	89,931	20,563	21,235	35.50	GLQ-1 (SWFU)	Yunnan, China	PP056124	Wang et al. 2024
***Angiopteris nodosipetiolata*** Ting Wang tris, H.F. Chen & Y.H. Yan	152,963	89,932	20,561	21,235	35.50	GLQ-2 (CSH)	Yunnan, China	PP056123	Wang et al. 2024
***Angiopteris sparsisora*** Ching	153,067	89,709	20,586	21,386	35.40	JLJ-09-0864 (HITBC)	Yunnan, China	PX138851	Jiang et al. 2025
***Angiopteris weimingii*** L.J. Jiang & Z.R. He	153,168	89,736	20,564	21,434	35.40	JLJ-0358-2 (HITBC)	Yunnan, China	C_AA132741	This study
***Angiopteris weimingii*** L.J. Jiang & Z.R. He	153,168	89,736	20,564	21,434	35.40	JLJ-09-0359 (PYU)	Yunnan, China	C_AA132740	This study
***Angiopteris weimingii*** L.J. Jiang & Z.R. He	153,168	89,736	20,564	21,434	35.40	JLJ-25-0612 (HITBC)	Yunnan, China	C_AA132742	This study
***Angiopteris yunnanensis*** Hieron	152,962	89,717	20,585	21,330	35.40	Liu-CP05 (HITBC)	Yunnan (Cult.), China	NC052844	Jiang et al. 2019

### Plastome assembly, annotation, and phylogenetic analyses

Complete plastomes were assembled using GetOrganelle v1.7.1 (Jin et al. 2020). Assembled plastomes were initially annotated using PGA (Qu et al. 2019) to detect and annotate all genes. All tRNAs were confirmed using tRNAscan-SE v2.0.7 (Chan and Lowe 2019). For further validation, the positions of start codons, stop codons, and introns were manually adjusted in Geneious Prime 2019.2.1. Sequences downloaded from GenBank and newly generated data were aligned in Geneious using the MAFFT v7.450 plugin (Katoh and Standley 2013) with the E-INS strategy. Poorly aligned regions were removed using Gblocks v0.91b (Talavera and Castresana 2007). ModelFinder was used to infer the most appropriate nucleotide substitution model based on the corrected Akaike Information Criterion (AICc). Maximum likelihood (ML) bootstrapping was conducted with 5,000 rapid bootstrap (BS) replicates in IQ-TREE v2.1.3 (Nguyen et al. 2015) in a single run. Bayesian inference (BI) was performed using MrBayes v3.2.2 (Ronquist et al. 2012) with two runs of four Markov chain Monte Carlo chains, each beginning with a random tree and sampling one tree every 1,000 generations over 2,000,000 generations. The standard deviation of split frequencies was below 0.001, and the Markov chain Monte Carlo output was examined to confirm convergence and ensure that all effective sample size (ESS) values exceeded 200. Maximum likelihood bootstrap support (ML-BS) values and Bayesian inference posterior probabilities (BI-PP) were generated and visualized using FigTree v1.4.3 (Rambaut 2017).

## Results

The complete plastome of the new species displayed a typical quadripartite structure, consisting of a pair of inverted repeats (IRA and IRB), separated by the large single-copy region (LSC) and the small single-copy region (SSC) (Table 1). The overall GC content of the plastome was 35.40%, and the lengths of the LSC, SSC, and IR regions were 89,736 bp, 20,564 bp, and 21,434 bp, respectively (Table 1). The concatenated dataset was 152,766 bp in length, of which 370 sites were parsimony informative. GTR+F+I was selected as the best model of evolution in both ML and BI analyses.

**Figure 1.**
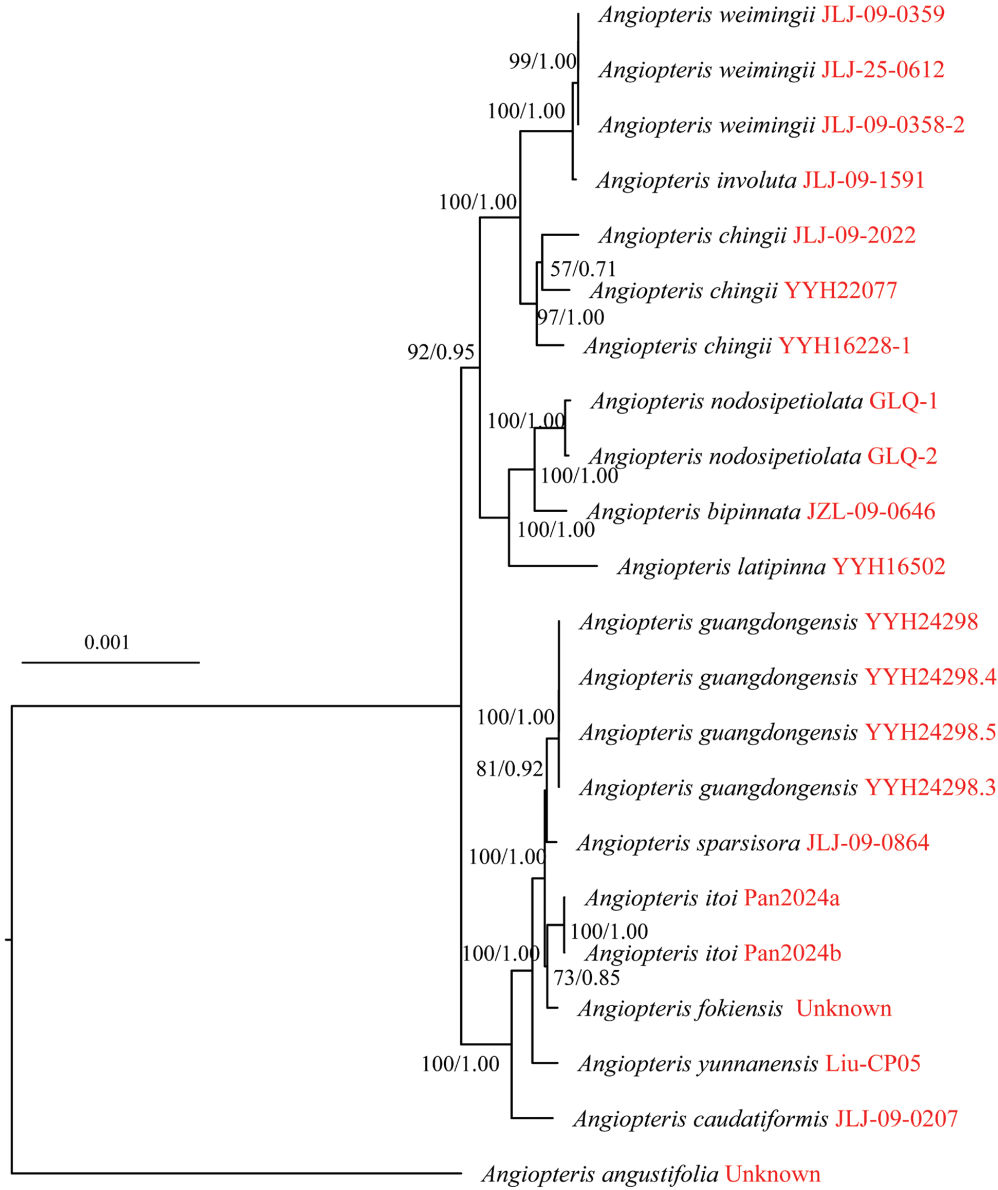
Maximum likelihood tree of *Angiopteris weimingii* and its putative closely related taxa based on complete plastome sequences. Maximum likelihood bootstrap support (ML-BS) and Bayesian inference posterior probability (BI-PP) values are shown above the branches.

Phylogenetic analyses based on complete plastome datasets revealed generally congruent topologies (Fig. 1). Except for *Angiopteris angustifolia*, the remaining species of *Angiopteris* could be divided into two clades: the *Angiopteris* clade (including *Angiopteris caudatiformis*, *A. fokiensis*, *A. guangdongensis*, *A. itoi*, *A. sparsisora*, and *A. yunnanensis*) and the *Archangiopteris* clade (including *A. bipinnata*, *A. involuta*, *A. latipinna*, *A. nodosipetiolata*, and *A. weimingii*). Within the *Angiopteris* clade, *A. caudatiformis* was resolved as sister to the remaining five species in our analysis. Within the *Archangiopteris* clade, *A. involuta* and *A. weimingii* formed a strongly supported monophyletic clade (ML-BS = 99, BI-PP = 1.0) and were sister to *A. chingii*. These three species together were sister to the clade comprising *A. bipinnata*, *A. latipinna*, and *A. nodosipetiolata*, with strong support (ML-BS = 92, BI-PP = 0.95).

**Figure 2.**
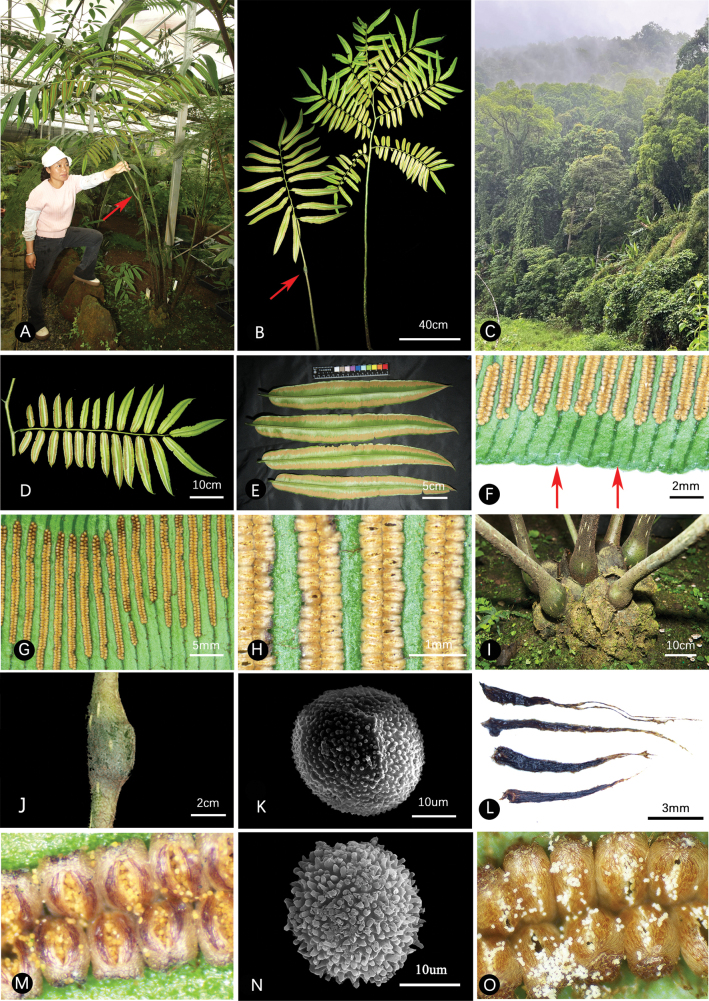
*Angiopteris weimingii* L.J.Jiang & Z.R.He. **A**. Cultivated individual (in XTBG). The red arrow indicates the pulvinate articulation at the stipe in pinnate fronds; **B**. Pinnate and bipinnate fertile fronds; **C**. Habitat; **D**. Pinna of a bipinnate frond; **E**. Pinnule; **F**. Leaf margin and retrograde false veins; **G**. Elongate linear sori; **H**. Mature sporangia; **I**. Erect rhizome; **J**. Pulvinate articulation at the middle-lower part of the stipe in pinnate fronds; **K**. Spores with densely verrucate ornamentation; **L**. Black scales on the stipe; **M**. *A. weimingii* mature sporangia releasing yellow spores; **N**. *A. itoi* spores with clavate-echinate ornamentation; **O**. *A. itoi* mature sporangia releasing white spores (materials from Wulai, Taiwan Province).

**Figure 3.**
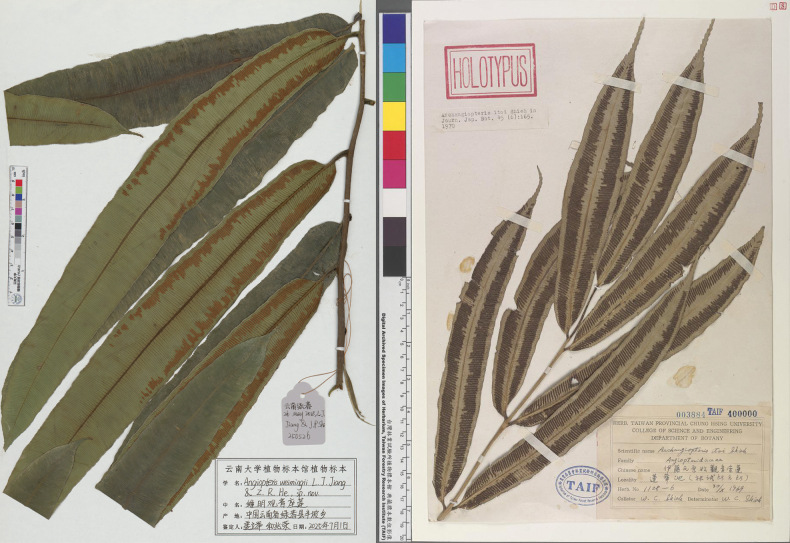
The type specimens of *Angiopteris weimingii* (left, PYU) and *A. itoi* (right, TAIF) clearly exhibit the acute pinna tips of the former, in contrast to the attenuate pinna tips of the latter.

*Angiopteris weimingii* is morphologically intermediate between the *Archangiopteris* clade and the *Angiopteris* clade, characterized by fertile once-pinnate and bipinnate fronds and long linear sori (Fig. 2). It is most morphologically similar to *A. itoi* from Taiwan Province, which was inferred as a hybrid between *A. lygodiifolia* and *A. somae* (Wu 2002), but differs by its taller plants, pinnae apices that are abruptly acuminate with a short caudate tip, sori positioned closer to the leaf margin, and shorter false veins (Fig. 3; Table 2). Despite their morphological similarity, plastid-based phylogenetic analyses revealed that *A. weimingii* and *A. itoi* reside in two distinct clades, with *A. weimingii* placed within the *Archangiopteris* clade and *A. itoi* within the *Angiopteris* clade. Spore morphology also provides a key diagnostic feature for distinguishing *A. weimingii* from *A. itoi* (Fig. 2K, M, N, O). The mature spores of *A. weimingii* are yellow and possess densely verrucate ornamentation, whereas those of *A. itoi* are white with clavate-echinate ornamentation. This distinct difference in both ornamentation type and spore color constitutes an important diagnostic characteristic. Morphological and molecular evidence collectively supports *A. weimingii* as a new species.

**Table 2.** Morphological comparison of *Angiopteris weimingii*, *A. involuta*, *A. sugongii*, and *A. itoi*

Characters	*A. weimingii*	*A. involuta*	*A. sugongii*	*A. itoi*
Frond	120–250 cm	60–120 cm	70–160 cm	50–150 cm
Stipe	45–140 cm, smooth	35–70 cm, slightly tuberculate	50–120 cm, smooth	30–80 cm, smooth
Rhizome	erect	ascending	ascending	erect
Laminae	Laminae once pinnate to bipinnate, pinnae 8–13 pairs, 8–45 × 2.5–5.5 cm, bases truncate or rounded, rarely cordate, margin crenulate, apex is abruptly acuminate with a short caudate tip (ca. 1 cm).	Laminae once pinnate to bipinnate, pinnae 5–7 pairs, 5–14 × 1.5–3 cm, bases cuneate, margin crenulate. The fertile leaf margins curl upward into a boat shape.	Laminae once pinnate to bipinnate, pinnae 5–12 pairs, 25–35 × 3–5.5 cm, bases cuneate, margin coarsely dentate.	Laminae once pinnate to bipinnate, pinnae 4–9 pairs, 26–34 × 3–5 cm, bases cuneate, margin undulate, apex is acuminate with a long caudate tip (2–4 cm)
False veins	present (0.5–2 mm)	absent (or obscure)	absent (or obscure)	obscure or present (longer than sori if present)
Sori	sori ca. 2 mm from margin, 0.2–1.5 cm, with 12–120 sporangia	sori ca. 1–2 mm from margin, ca. 0.2–0.8 cm, with 14–60 sporangia	sori ca. 1–2 mm from margin, ca. 0.2–1.2 cm, with 20–80 sporangia	sori medial, ca. 3–5 mm from margin, 0.2–1.2 cm, with 12–90 sporangia
Exospores	verrucose ornamentation	tuberculate-spinose ornamentation	spiculose ornamentation	clavate-echinate ornamentation
Distribution	Lvchun, Yunnan, China	Jiangcheng, Yunnan, China	Hekou, Yunnan, China	Lianhuachi and Wulai, Taiwan, China
References	This study	Jiang et al. 2025	Wang et al. 2021	Shieh 1970; Hsieh 2008; Guo 2020

## Discussion

*Angiopteris weimingii* offers novel insights into the taxonomy of *Angiopteris*. Molecular phylogenetic studies have consistently placed the *Archangiopteris* clade within *Angiopteris* (Li and Lu 2006; Wang et al. 2024). Although *A. weimingii* is morphologically allied with *A. itoi*, which belongs to the *Angiopteris* clade, it is nested within the *Archangiopteris* clade (Fig. 1). This finding supports the taxonomic merger of the two genera and underscores the remarkable evolutionary complexity within *Angiopteris*. We further hypothesize that *A. weimingii* may represent a hybrid between *Angiopteris* and a typical *Archangiopteris* lineage. Given that both *A. sparsisora* and *A. itoi* are presumed to be hybrids (Ching and Wang 1982; Wu 2002), this hypothesis warrants validation through future integrative studies.

## Taxonomy

### Angiopteris weimingii

Taxon classificationPlantaeMarattialesMarattiaceae

L.J.Jiang & Z.R.He sp. nov.

6574177C-CBDE-569C-9B18-EE49FDE804A1

urn:lsid:ipni.org:names:77376164-1

Fig. 2

#### Type.

China • Yunnan: Lvchun County, Banpo Township, 26 May 2025, *L.J. Jiang & J.P. Shi JLJ*-*09-0359* (Holotype: PYU JLJ-250526!; Isotype: HITBC C090370!).

#### Diagnosis.

*Angiopteris weimingii* (endemic to Yunnan) is most similar morphologically to *A. itoi* (endemic to Taiwan), but differs in the following key characters: taller plants (up to 2.5 m), pinnule apex with a short caudate tip (ca. 1 cm), sori positioned 2 mm from margin, mature spore color is yellow and possess a densely verrucate ornamentation. In contrast, *A. itoi* is shorter (ca. 1.5 m), pinnule apex with a long caudate tip (2–4 cm), mature spore color is white with clavate-echinate ornamentation. Phylogenetic analysis revealed that *A. weimingii* is most closely related to *A. involuta*, yet *A. weimingii* is notably larger (often exceeding 1.2 m), with a smooth stipe and retrograde false veins. However, *A. involuta* is typically under 1.2 m, has tuberculate stipes, lacks retrograde false veins, and possesses consistently enrolled fertile pinnae. Comparative morphology with related species is provided in Table 2.

#### Description.

**Plants** terrestrial, 120–250 cm tall. **Rhizome** stout, erect. **Fronds** clustered, once pinnate to bipinnate. **Stipes** 45–140 cm long, terete, 1–1.5 cm in diameter, smooth; densely covered with brown filamentous hairs when young (caducous), sparsely scaly with black, narrowly lanceolate scales (ca. 0.05–0.1 × 0.5–2 cm). **Lamina** up to 250 cm long. **Pinnae** once pinnate to bipinnate, pinna bases truncate or rounded, rarely cordate, margins crenate–serrulate and undulate, pinnule apex acute with a short caudate tip (ca. 1 cm). **Venation**: Veins simple or forked, bearing acroscopic false veins (ca. 0.5–2 mm long, never extending to sori position). **Texture** herbaceous, adaxially green, abaxially pale green when dry. **Sori** linear, 0.2–1.5 cm long, composed of 12–120 sporangia, positioned 2 mm from margin. **Spores** yellow, trilete; exospores densely tuberculate. The spore matures from May to July.

#### Etymology.

The specific epithet “weimingii” is designated in honor of Prof. Wei-Ming Chu, an esteemed Chinese pteridologist, in recognition of his contributions to the genus *Angiopteris*. The Chinese name is suggested as ‘维明观音座莲 (wei ming guan yin zuo lian)’.

#### Distribution and habitat.

Endemic to southern Yunnan (Lvchun County) and grows in rubber plantations understory along streams, alt. 270–350 m.

#### Conservation status.

The species inhabits the understory of rubber plantations, where anthropogenic disturbance is extremely severe. Only one population (consisting of approximately 30 plants) is found in the wild. In accordance with the IUCN (2024) standards, the species is tentatively designated as **Critically Endangered (CR)**, and further investigations in similar forests are still needed.

#### Additional specimens examined.

China • Yunnan: Lvchun County, Banpo Township, 26 June 2025, *JLJ-09-0358-2* (HITBC!); *JLJ-25-0612* (HITBC!).

##### Keys to the species with fertile fronds in both once-pinnate and bipinnate laminae of *Angiopteris* in China and surrounding regions

**Table:** 

1	Sori short-linear, usually less than 5 mm long, marginal	**2**
–	Sori long-linear, usually more than 5 mm long, medial (on the veinlets)	**4**
2	Rhizome creeping. Sori ca. 3–4 mm long, positioned 3–4 mm from margin	***A. sparsisora***
–	Rhizome erect. Sori ca. 1 mm long, positioned 0.5–1 mm from margin	**3**
3	Stipe smooth; pinnules lanceolate	***A. caudatiformis***
–	Stipe with transversely raised stripes; pinnules oblanceolate, apex abruptly acute with a short caudate tip	***A. amamensis***
4	Rhizome erect	**5**
–	Rhizome ascending	**6**
5	Plants height not exceeding 150 cm; pinnae 4–9 pairs, pinnule apex attenuate, false veins absent or present; sori positioned 3–5 mm from margin, mature spores color is white with clavate-echinate ornamentation (Taiwan)	***A. itoi***
–	Plants height usually exceeding 150 cm, up to 250 cm; pinnae 8–13 pairs, pinnule apex acute, false veins short (ca. 0.5–2 mm, never reaching soral position); sori positioned 2 mm from margin, mature spores color is yellow with verrucose ornamentation (Yunnan)	***A. weimingii***
6	Plants small, mature fronds usually < 1 m; veins sparse, 5–6 per cm; leaf margins coarsely serrate; sori ca. 4 mm from margin	***A. bipinnata***
–	Plants large, mature fronds > 1 m; veins dense, 8–10 per cm; leaf margins undulate or minutely serrate; sori ca. 1–2 mm from margin	**7**
7	Stipes smooth, petiole scales reddish brown; fertile pinnules flat; exospores with spiculose ornamentation	***A. sugongii***
–	Stipes sparsely tuberculate, petiole scales black or dark brown; fertile pinnules with revolute margins (boat-shaped); exospores with densely tuberculate-spinose ornamentation	***A. involuta***

## Supplementary Material

XML Treatment for Angiopteris weimingii

## Additional information

### Use of AI

No use of AI was reported.

### Author ORCIDs

Li-Ju Jiang https://orcid.org/0009-0006-2145-3836

Jing Zhao https://orcid.org/0000-0001-7871-2209

Dan-Ni Ma https://orcid.org/0009-0002-0948-1536

Jian-Wu Li https://orcid.org/0000-0003-2410-0666

Xin-Mao Zhou https://orcid.org/0000-0003-3555-7784

Jia-Guan Wang https://orcid.org/0000-0003-0264-9180

Zhao-Rong He https://orcid.org/0000-0001-6492-3689

### Data availability

All of the data that support the findings of this study are available in the main text.
